# Lea’s Series of Medical Epitomes

**Published:** 1905-09

**Authors:** 


**Lea's Series of Medical Epitomes.** Edited by **Victor C. Pedersen,** M.D. Clinical Diagnosis and Uranalysis. A Manual for Students and Practitioners. By James R. Arneill, A.B., M.D., Professor of Medicine and Clinical Medicine in the University of Colorado, Denver. Duodecimo, 244 pages, with 79 engravings and a colored plate. Philadelphia and New York: Lea Brothers & Co. 1905. ($1.00, net.)

It is seldom that so much really valuable and useful material is crowded into a small book as is contained in this number of the Medical Epitome Series. It covers clinical examination of blood, its study, and pathologic blood conditions; the stomach, feces, sputum, urinalysis and the examination of urinary sediments. There is a completeness which is satisfactory in every subject presented and every possible help is given in advice as to equipment, reagents and stains. The section devoted to urinalysis is particularly clear and comprehensive and each step is so clean-cut that error is practically impossible. The book is excellently illustrated. N. W. W.

